# The effect of a phosphorus-based FR on the fire performance and flammability properties of basalt fiber-reinforced acrylonitrile-butadiene-styrene composites

**DOI:** 10.55730/1300-0527.3473

**Published:** 2022-08-18

**Authors:** Çağrıalp ARSLAN, Mehmet DOĞAN

**Affiliations:** 1 Department of Textile Engineering, Faculty of Engineering, Architecture and Design, Bartin University, Bartın, Turkey; 2 Department of Textile Engineering, Faculty of Engineering, Erciyes University, Kayseri, Turkey

**Keywords:** Acrylonitrile-butadiene-styrene, basalt fiber, aluminum diethyl phosphinate, composite, flame retardancy, fire performance

## Abstract

This study focuses on the improvement in fire performance, and flame retardancy (FR) properties of chopped basalt fiber-reinforced acrylonitrile-butadiene-styrene (ABS) composites. For this purpose, different amounts of aluminum diethyl phosphinate (AlPi) compound (5, 10, and 15 wt%) were incorporated in the composites. The FR properties of the composites were examined via limiting oxygen index (LOI), UL-94 standard, and mass loss calorimeter tests. Thermogravimetric analysis was carried out to analyze the decomposition behavior of the composites. SEM inspection was also performed to examine the char surfaces of the composites. The results and findings showed that the introduction of AlPi compound into the composite structure leads to promotion in the char yield and improves the fire performance of the ABS matrix. As the added amount of AlPi into the composite increased, the LOI value of the composite increased. The addition of 15 wt% AlPi resulted in a UL-94 rating of V1 and the LOI value of 31.4%.

## 1. Introduction

Acrylonitrile-butadiene-styrene (ABS) is one of the most widely used thermoplastic terpolymers in industrial-based applications. Especially its low cost, good toughness, easy processability, and chemical resistance make it attractive for electrical and automotive industries [1–3].

On the other hand, the easy flammability feature of ABS emerges as one of the main handicaps, besides all these promising properties [1, 2]. Various halogen-containing and halogen-free flame retardants are applied to the ABS to overcome this problem. Although the most effective flame retardants (FR) are halogen-containing compounds used in the ABS, their environmentally hazardous effects highlight the use priority of halogen-free FR compounds, such as phosphorus-, silicon-, nitrogen-, and other elements-based [2]. In the literature, phosphorus-based compounds are the most commonly used, halogen-free FR chemicals in the ABS, including aluminum hypophosphite (AHP) [2, 4–9], aluminum diethyl phosphinate [9, 29], ammonium polyphosphate (APP) [1, 4, 10–19], red phosphorus (RP) [14], diphenyl phosphate [20, 21], DOPO derivatives [21–24], aryl-phosphates compounds [25, 26], phosphorus-nitrogen containing phenol formaldehyde [27], nitrogen-containing alkylphosphinate [28], piperazine pyrophosphate [29], and melamine phosphate [30].

The noncombustible inorganic fibers, such as basalt (BF) and glass fibers, show a “wick effect” that increases the flammability of the polymer matrix. However, the wick effect for the chopped or milled fiber remains low due to discontinuing fiber paths, compared to their continued form [31]. Only a few studies on the FR properties of different polymer composites containing the BF are found in the literature [32–37]. In these studies, the BF is used with a biodegradable starch resin [32], HDPE [33], PLA [34], PA6 [35], cyanate ester/epoxy blended polymers [36], EVA [37, 38], PP [39], epoxy [40]. The RP powder and Mg(OH) [32], AHP [34] and nickel alginate-brucite-based FR [37] halogen-free compounds are used to improve the FR properties of the composites.

In our previous study, (3-Aminopropyl) triethoxysilane (AP) modified BF (AP-BF) is compounded with ABS matrix, and the mechanical performance of composites is analyzed [41]. In the current study, aluminum diethyl phosphinate (AlPi) compound at different concentrations is used to improve the FR properties of AP modified chopped BF reinforced ABS composites. The novelty of this study is that, to the best of our knowledge, it is the first study to investigate the FR properties of BF reinforced ABS composites containing AlPi. The FR properties of the composites are analyzed through the limiting oxygen index (LOI), UL-94 standard, and mass loss calorimeter tests. The decomposition behavior of the composites is investigated via thermogravimetric analysis (TGA). The SEM analysis is performed to inspect the char surfaces of the composites.

## 2. Materials and methods

### 2.1. Materials

ABS natural terpolymer, which has a grade of HI121H and a density of 1.04 g/cm^3^, was purchased from LG Chem. BF, having a density of 2.8 g/cm^3^, was supplied from Tila Kompozit with a chopped length of 6 mm and a diameter of 13–20 **μm.** (3-Aminopropyl) triethoxysilane (AP) modified BF was used in the study. The AP, having a density of 0.946 g/cm^3^ and 221.37 g/mol molecular weight, was supplied from Sigma Aldrich. The aluminum diethyl phosphinate (AlPi) has a phosphorus content of 19.5–20.5 wt%, a density of 1.3 g/cm^3^, and a decomposition temperature of >350 **°**C with a trading name of Exolit® OP 950, which is an organic phosphinate-based FR. It was purchased from Clariant.

### 2.2. Methods

The mixtures containing various composition ratios were prepared via a laboratory-type corotating twin-screw extruder (Gülnar Makina, **İ**stanbul, Turkey) (L/D: 40; Φ: 16 mm) with a temperature profile of 50-190-200-210-210-210 **°**C at 100 rpm. The specimens for the flammability tests were molded in a laboratory-scale injection-molding machine DSM Xplore 12 mL Micro-injection Molder, Netherlands) at a barrel temperature of 235 **°**C and a mold temperature of 32 **°**C. The specimens for the mass loss calorimeter test were produced on a laboratory scale hot press (Gülnar Plastics Machines, **İ**stanbul, Turkey) at 185 **°**C for 210 s.

The following procedure was applied for the production of composites: the BF amount in the composites is kept constant (20 wt%). Three different amounts of AlPi (5, 10, and 15 wt%) containing BF reinforced ABS composites were prepared. The sample codes and compositions are given in Table 1. The loading level of 20% for BF was preferred since the optimum performances were achieved for that filling ratio in our previous study [41].

The TGA tests were performed using a Perkin Elmer Diamond instrument on the specimens under a nitrogen atmosphere (50 mL/min) from room temperature to 800 °C with a 10 °C/min heating rate. The LOI measurements were conducted on Fire Testing Technology Limiting Oxygen Index Analyzer instrument using the specimens having the nominal dimensions of 3.2 × 6.5 × 130 mm^3^, according to ASTM D2863. UL-94V vertical burning tests were also implemented to examine the flammability properties of the composites using the specimens with the dimensions of 13 × 130 × 3.2 mm^3^, according to the ASTM D3801. Mass loss calorimeter tests were performed using Mass Loss Cone with thermopile attachment (Fire testing Technology, UK), and the procedures in the ISO 13927 standard were followed. The square-shaped specimens with the dimensions of 100 × 100 × 3 mm^3^ were used. Corresponding to a mild fire scenario, they were irradiated at a heat flux of 35 kWm^−2^. The char surfaces of the composites after the mass loss calorimeter test were inspected via SEM, ZEISS EVO LS10 computer controlled digital instrument and accelerating voltage of 20 kV. The specimens were sputter-coated with Au/Pd alloy before the analysis.

## 3. Results and discussion

### 3.1. Thermal decomposition

**Figure 1:**
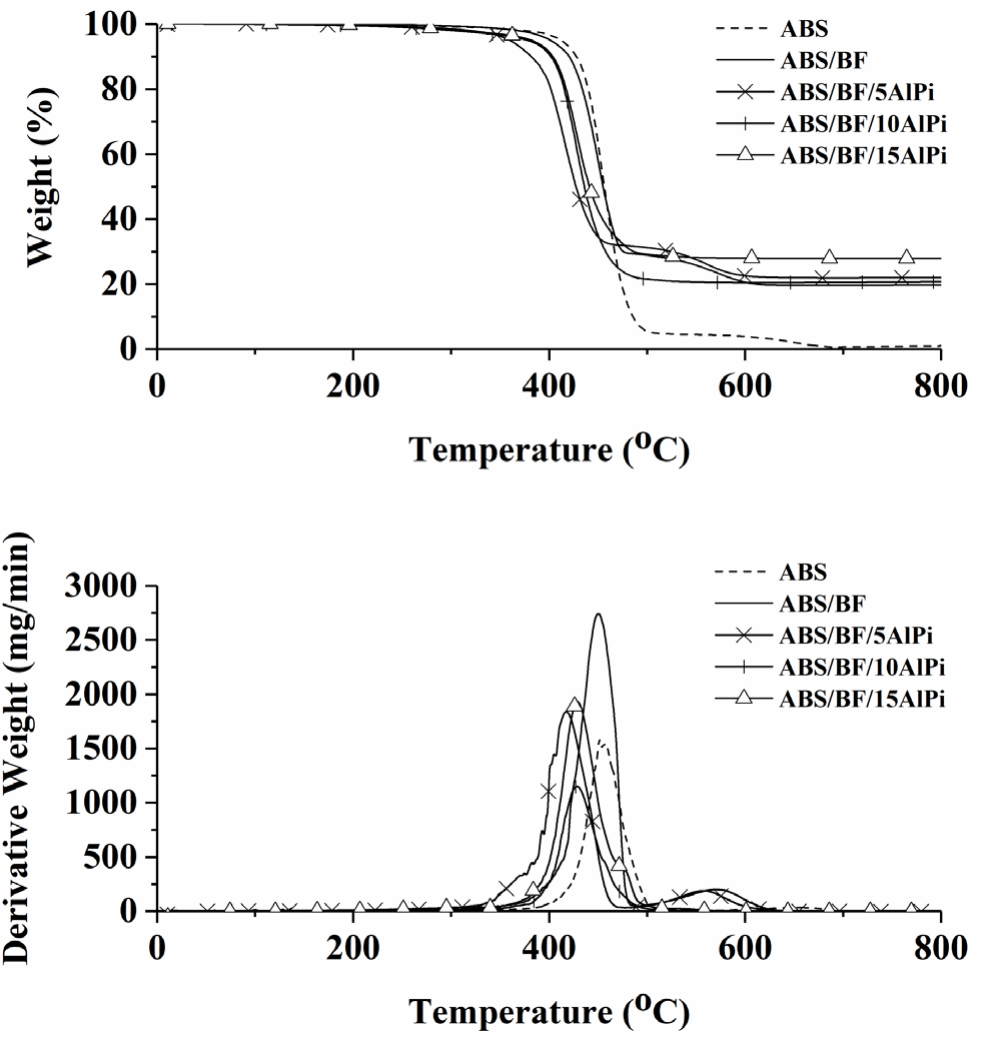
TGA and DTG curves of the composites.

TGA tests are performed to analyze the thermal stability of pristine ABS and the relevant composites. The TGA and DTG curves are shown in Figure 1 and the relevant data are listed in Table 2. As seen from the Figure 1 and Table 2, the thermal decomposition of ABS occurs in two steps with the onset (T_1%_), and two maximum (T_max1_ and T_max2_) decomposition temperatures of 321, 451, and 653 °C, respectively. The ABS leaves a char residue yield of 1.3% at the end of the test. In the first decomposition step, the ABS terpolymer undergoes structural decomposition [42–44]. Along with, it is thought that the second decomposition step may stem from the decomposition of residual char [45]. The addition of BF into the ABS enhances the initial thermal stability (T_1%_), whereas it causes a drastic fall in T_max2 of the ABS while the_ T_max1_ remains almost the same. The undecomposed nature of BF is directly effective on an extreme increase in the char yield.

A remarkable decrease is seen in the thermal stability of the ABS matrix with the incorporation of AlPi regardless of its amount. Thermal decomposition of the composites containing AlPi happens in two steps for the composite with 5 wt% AlPi and a single step for the further concentration.

In the literature, it is stated that the AlPi undergoes thermal degradation in two steps. The first step occurs at 340–550 °C, and the AlPi loses a large part of its mass in this temperature range. The second step occurs at 850–1000 **°**C with a very small mass loss [9, 46–48]. However, it is seen that the second step decomposition temperature for AlPi exceeds the range of test temperature. Therefore, no T_max2_ data can be obtained for the composites having AlPi, except for 5 wt% concentration. For the composites having the AlPi, the first step maximum decomposition occurs at around 420 **°**C, which stems from partial vaporizing of AlPi and partial decomposition of AlPi to diethyl phosphinic acid (C_4_H_11_O_2_P) and CH_3_ [9, 46–48]. The addition of 15 wt% AlPi significantly increases the char yield of the composite to 28.0%, indicating that the higher concentration of AlPi over the 10 wt% has a promotion effect on the char formation of ABS. From the TGA test results, the incorporation of AlPi compound has a positive effect on the char formation, whereas it reduces the thermal stability of ABS matrix.

### 3.2. Flammability properties

The flammability characteristics of the composites are determined by LOI value and UL-94 ratings. The relevant test data are given in Table 2. ABS resin has an LOI value of 18.5% and burns to clamp during the UL-94 test. The LOI value of the ABS reduces with the addition of inorganic BF as the UL-94 rating does not change. The BF has the “candlewick effect,” which causes the increase in flammability of polymeric matrix in the combustion process [31]. In the literature, similar behavior is observed with BF and glass fiber in different matrix systems [31, 34, 49].

The LOI value of the composite increases with the incorporation of AlPi compound. As the added amount of AlPi into the composite increases, the LOI value of the composite increases. Increasing char yields and LOI values leads to improvement of the FR properties of the polymers [50, 51]. A meaningful improvement on the flammability properties of BF reinforced ABS composite is obtained with the use of AlPi at 15 wt % concentration. On the other hand, the composite containing a 5wt% amount of AlPi also fails in the UL-94 test. When the added amount of AlPi reaches 10 wt%, the LOI value of the composite remarkably increases, and the UL-94 rating shifts to V2. The best UL-94 rating (V1) and LOI value (31.4%) is achieved with the addition of 15 wt% AlPi.

### 3.3. Fire performance of the composites

**Figure 2:**
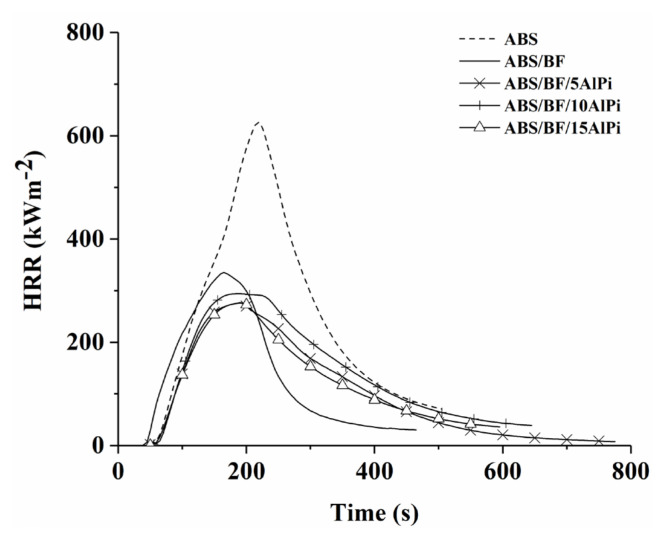
HRR curves of the pristine ABS and the relevant composites.

Fire performance of pristine ABS and the related composites are investigated via mass loss calorimeter tests. The peak heat release rate (pHRR), average heat release rate (aHRR), peak mass loss rate (pMLR), and total heat evolved (THE) are considered evaluation parameters for the fire performances of the composites. The lower value in these data means that better fire performance is achieved. The HRR curves of the pristine ABS and the related composites are shown in Figure 2. The relevant mass loss calorimeter data are listed in Table 3. According to Figure 2 and the tabulated values, pristine ABS burns very fast and almost completely after ignition with a char formation of 2.3%. A sharp HRR peak is observed with the pHRR value of 626 kWm^−2^. The addition of BF leads to a remarkable declining in the pHRR, aHRR, and THE values and increase in the residue owing to noncombustible characteristics of the BF. The BF shows a barrier effect against the combustible gases and oxygen penetration into the burning polymer; thus, it enhances the fire performance of the ABS matrix. The presence of the FR compounds into the ABS matrix causes the shape of HRR curves of ABS and ABS/BF composite to shift to plateau-like and expands the burning time of the ABS. When a large part of BF retaining its weight during the test according to the ABS/BF with residue yield of 22.7 wt% is considered, no proportional results are obtained in the residue yields of the composites containing FR compounds.

Another notable point is that while less char yield in TGA and char residue in MLC test are obtained, higher LOI, pHRR, pMLR, and THE values are obtained with the use of 10 wt% AlPi comparing to the addition of 5 wt%, contrary to expectation. This can be attributed to the fact that phosphorus based FR compounds such as AlPi exhibit different behavior in air and inert atmospheres because TGA is a test method performed under a nitrogen atmosphere, and the LOI and Mass Loss Calorimetry tests are performed in air atmosphere.

Regardless of the amount, the AlPi compound reduces the pHRR, aHRR, and pMLR values of the ABS/BF composite. In addition, more flat HRR curves and delayed burning times are observed using AlPi. The addition of 5 and 15 wt% AlPi reduces the pHRR value of composite by about 17%. The AlPi shows its FR effect in the gas phase as a flame inhibitor via Lewis acid-base interaction with the polymer [46, 47].

The pristine ABS has THE of 104 MJ/m^2^. This value decreases to 78 MJ/m^2^ with the addition of BF. The composites with the AlPi show lower THE values than the corresponding value of the ABS/BF composite. A significant reduction is achieved in THE value of ABS/BF composite using 5 and 10wt% AlPi. The lowest THE (64 MJ/m^2^) is obtained with the AlPi loading of 15wt%. The decrease in THE value is due to the increment in char residue and means that the composite presents better fire performance.

THE/TML value is an indicator of effective combustion heat. The reduction in this value indicates that FR compounds show action mode in the gas phase through the flame inhibition. The THE/TML value for pristine ABS is 3.22 MJ/g^2^, and this value remains almost the same with the introduction of BF into ABS. A remarkable decrease in THE/TML value is observed with the use of AlPi compared to pristine ABS and ABS/BF composite. This effect is observed in all AlPi concentrations. The lowest THE/TML (2.41 MJ/g^2^) is obtained with the AlPi loading of 5wt%. It indicates that the AlPi has a prominent mode of action on the gas phase.

**Figure 3:**
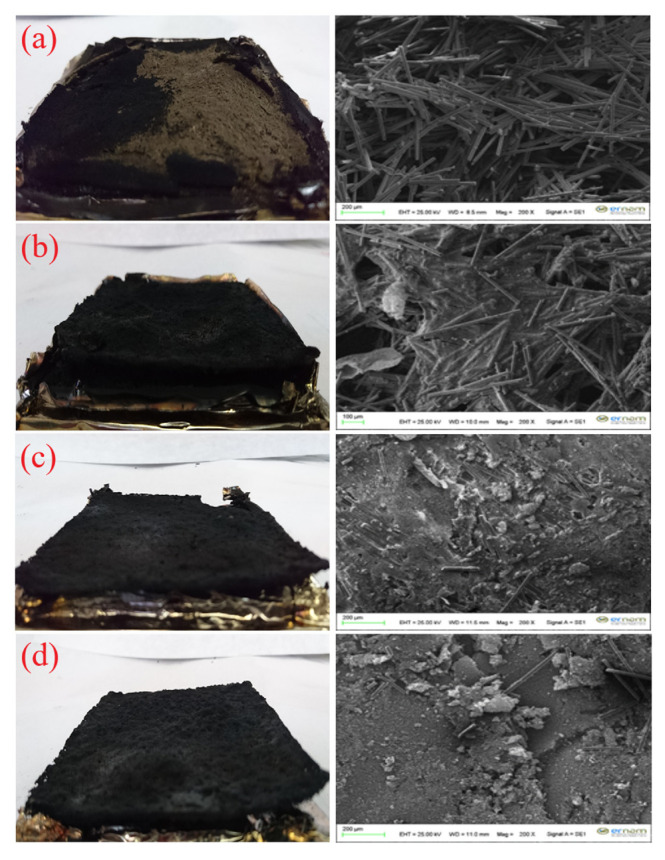
Digital photographs (left side) and SEM micrographs (right side) at magnifications of 200× of the char residues remaining after mass loss calorimeter test: a) ABS/BF, b) ABS/BF/5AlPi, c) ABS/BF/10AlPi, and d) ABS/BF/15AlPi.

The char residues that remained after the mass loss calorimeter test are inspected to understand the composites’ FR mechanism, structure, and morphology. The digital photographs and SEM micrographs of the char residues remained after the mass loss calorimeter test of ABS/BF and ABS/BF/AlPi composites are shown in Figure 3. From the digital photographs of char residues, the remained BF, having undecomposed characteristic, in ABS/BF composite can be easily seen in green color. According to the SEM images, very few solid chars are observed around the BFs. All the composites containing AlPi presents a porous and thinner crisp char layer. However, the number of the porous structure decreases as the added amount of AlPi increases. No remarkable difference is observed between the char residue of the composites at the loading ratio of 10 and 15 wt% AlPi, while the composite containing 5 wt% AlPi presents more of and bigger porous structures.

## 4. Conclusions

In this study, the effect of the aluminum diethyl phosphinate (AlPi) compound and its concentration on the FR properties of silane-modified BF reinforced ABS composite is studied. The composites were characterized with thermogravimetric analysis, LOI, UL-94 V, and mass loss calorimeter tests. According to the TGA test results, the incorporation of the AlPi compound into the composite structure causes promotion in the char yield, whereas a remarkable decrease is observed in the thermal stability of the ABS matrix, regardless of its amount. When the mass loss calorimeter test results of the composites are evaluated, the addition of BF enhances the fire performance of the ABS matrix via the barrier effect against the combustible gases and oxygen penetration. The composites containing the AlPi have lower pHRR, aHRR, and pMLR values than the corresponding values of ABS/BF composite. The addition of 5 and 15 wt% AlPi reduces the pHRR value of composite by about 17%. According to the flammability test results, the addition of BF reduces the LOI value of ABS due to the “candlewick effect”. On the other hand, as the added amount of AlPi into the composite increases, the LOI value of the composite increases, while the highest LOI value is achieved with the use of 15 wt% AlPi. The best UL-94 rating (V1) and LOI value (31.4%) is achieved with the addition of 15 wt% AlPi.

## Figures and Tables

**Table 1:** The sample codes and compositions.

Sample Code	ABS (wt. %)	BF (wt. %)	AlPi (wt. %)
ABS	100	0	0
ABS/BF	80	20	0
ABS/BF/5AlPi	75	20	5
ABS/BF/10AlPi	70	20	10
ABS/BF/15AlPi	65	20	15

**Table 2:** The TGA test data, LOI values, and UL-94 ratings of the composites.

Sample	TGA				LOI (%)	UL-94 V
	T_1%_ (°C)	T_max1_ (°C)	T_max2_ (°C)	Char Yield at 800 °C (%)		
ABS	321	451	653	1.3	18.5	BC
ABS/BF	332	450	571	19.8	17.8	BC
ABS/BF/5AlPi	233	418	559	22.0	22.4	BC
ABS/BF/10AlPi	250	428	-	20.8	26.5	V2
ABS/BF/15AlPi	265	429	-	28.0	31.4	V1

* BC**:** burn to clamp

**Table 3:** Mass loss calorimeter test data of the composites.

Sample	Test Duration (s)	pHRR	aHRR	pMLR	THE	THE/TML	Residue (%)
ABS	518	626	323	0.25	104	3.22	2.3
ABS/BF	481	335	229	0.31	78	3.21	22.7
ABS/BF/5AlPi	788	277	147	0.18	70	2.41	25.9
ABS/BF/10AlPi	645	294	176	0.25	79	2.52	18.8
ABS/BF/15AlPi	602	276	153	0.22	64	2.46	23.2

* pHRR: peak heat release rate (kW/m^2^), aHRR: average HRR (kW/m^2^), pMLR: peak mass loss rate (g/s), THE: total heat evolved (MJ/m^2^), TML: total mass loss (g).
